# Tools to Perform Local Dense 3D Reconstruction of Shallow Water Seabed ‡

**DOI:** 10.3390/s16050712

**Published:** 2016-05-17

**Authors:** Loïca Avanthey, Laurent Beaudoin, Antoine Gademer, Michel Roux

**Affiliations:** 1 Laboratoire LTCI, CNRS, Télécom ParisTech, Université Paris-Saclay, 46 rue Barrault, 75013 Paris, France; michel.roux@telecom-paristech.fr; 2 ESIEA, 9 rue Vésale, 75005 Paris, France; dr.beaudoin@gmail.com; 3 EPF, 21 Boulevard Berthelot, 34000 Montpellier, France; antoine.gademer@epf.fr

**Keywords:** underwater 3D reconstruction, dense point clouds, low cost underwater micro-robot, stereoscopic rig

## Abstract

Tasks such as distinguishing or identifying individual objects of interest require the production of dense local clouds at the scale of these individual objects of interest. Due to the physical and dynamic properties of an underwater environment, the usual dense matching algorithms must be rethought in order to be adaptive. These properties also imply that the scene must be observed at close range. Classic robotized acquisition systems are oversized for local studies in shallow water while the systematic acquisition of data is not guaranteed with divers. We address these two major issues through a multidisciplinary approach. To efficiently acquire on-demand stereoscopic pairs using simple logistics in small areas of shallow water, we devised an agile light-weight dedicated system which is easy to reproduce. To densely match two views in a reliable way, we devised a reconstruction algorithm that automatically accounts for the dynamics, variability and light absorption of the underwater environment. Field experiments in the Mediterranean Sea were used to assess the results.

## 1. Introduction

The medium term objective of this study is to develop a complete system dedicated to precise thematic mapping of shallow underwater areas, from data acquisition to three-dimensional reconstruction. Intended to be used by field experts without relevant engineering skills, the designed system must meet the challenges posed by the study environment and provide comparable and reproducible three-dimensional data on demand. For this, it has to be light-weight, agile, affordable, easy to reproduce and highly automated.

This article starts with a survey of prior studies about mapping underwater environment in three dimensions and their limitation in the specific context of optical imagery in shallow water. It then presents the current state of the project and focuses on two majors issues. The first is the efficient acquisition of on-demand stereoscopic pairs in small areas of shallow water using simple logistics. The second is the reliable three-dimensional reconstruction from two views despite the dynamics of the environment, the highly variable quality of the images and strong light absorption. In the last part, we present the results obtained from field experiments in the Mediterranean Sea to assess our solutions.

## 2. Mapping the Underwater Environment in Three Dimensions

Three-dimensional mapping of submarine and subaquatic environments is important for many applications such as security [1], industry [2], environment [3] or archeology [4]. Underwater sensors for acquiring such data can mainly be grouped into two categories: acoustic and optical.

Those built on acoustic technology, such as sonars, are by far the most used. They can be used over large distances as sound wave propagation underwater takes advantage of the high elasticity of the medium [5,6]. Optical-based underwater sensors have to work closer to the observed scene due to poor electromagnetic wave propagation underwater [7], thus providing a smaller swath. However, they allow the acquisition of dense, multispectral and high resolution three-dimensional data. Optical-based sensors can be active like LiDARs (time-of-flight sensors) and laser scanners (active triangulation, structured light sensors) [8,9,10,11,12,13,14] or optical cameras (passive triangulation, temporal, binocular or multiviews sensors). The sensors used in this study belong to the latter category.

### 2.1. Acquisition Systems for Optical Imagery: Mobile Sensors

If we consider the platforms dedicated to optical imagery in shallow water (≲100 m deep) in the literature, we identify towed vehicles [15,16] or robots [17,18,19,20,21,22,23,24,25,26,27,28,29,30,31,32]. The robotic vehicles are either autonomous (AUV–*Autonomous Underwater Vehicle*) or wire-guided (ROV–*Remotely Operated Vehicle*) [33]. Examples of AUVs are SeaBED (*Woods Hole Oceanographic Institute*) and its derivatives, Girona500 and Sparus (*Universitat de Girona*), DAGON (*Deutsches Forschungzentrum für Künstliche Intelligenz*), Oberon (*Australian Centre for Field Robotics*), Starbug (*Commonwealth Scientific and Industrial Research Organisation*) or Aqua (*Dalhousie, MacGill and York Universities*). Lightweight ROVs include vehicles such as Phantom series (*Deep Ocean Engineering*) or V8 series (*Ocean Modules*).

Apart from the Aqua prototype, these platforms, designed for greater depths, are most of the time oversized when dealing with small, shallow water study areas (≲2000 m${}^{2}$) and often involve heavy logistics [25]. For these operational reasons, scientific work in shallow water mostly resorts to divers [34,35,36,37,38,39,40,41]. However, in doing so, the systematic nature of the automatic acquisition is lost, despite its importance for data completeness or acquisition process reproducibility. Recently, a new generation of micro-ROV has appeared, such as OpenROV or VideoRay, but these are currently mainly designed as wire-guided cameras rather than systematic acquisition systems and they do not yet have the modularity needed to conduct advanced scientific work.

In addition, native stereoscopic optical payloads (such as the BumbleBee of Point Grey) are rare and seldom allow changes in lens or baseline. Most of the time, rigs are built with professional sensors (from TriTech, Kongsberg or DeepSea P&L for example) mainly developed for the offshore petroleum industry. Their prices and sizes are in accordance with the great depths for which they were designed. Most other sensors used are compact or reflex cameras (from Canon, Nikon or GroPro for example) that are not designed to be easily controlled by a robotic system.

### 2.2. Processing Systems for Optical Imagery: Three-Dimensional Reconstruction Algorithms

**Figure 1:**
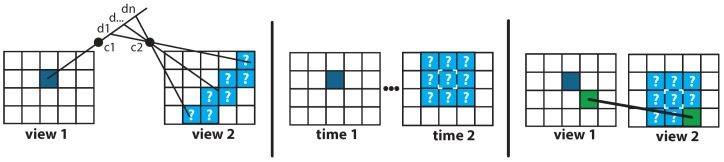
Diagrams representing the three strategies to reduce the complexity of the dense matching problem:geometric (**left**); spatio-temporal (**center**) and spatial neighborhood (**right**). The dark blue square represents the pixel whose counterpart in the other view is being sought; light blue represents the reduced search space defined by the criterion; in the right hand strategy green pixels are known correspondences.

Underwater studies have mostly been carried out to produce sparse point clouds [17,24,27,29,30,36,42,43,44,45,46] where the density is interpolated by meshing and texturing. These studies have mainly focused on the problem of registering these local clouds to obtain a model of the whole study area. Conversely, dense reconstruction algorithms directly produce point clouds with a finer granularity. As they seek to match all pixels of each view instead of a few sets of pixels, their complexity is much higher. This is called the *dense matching problem*. Strategies have been proposed to reduce the space of candidates and avoid the use of brute-force algorithms, thus making this problem computationally solvable. We have classified them in three categories: geometric, spatio-temporal and spatial neighborhood (see Figure 1):

- The geometric criterion exploits the epipolar constraint that links two stereoscopic views to reduce the search of a corresponding pixel to a line [47,48].
- The spatio-temporal criterion treats disparity as a slight temporal movement and therefore the corresponding pixel is sought near its original position in the first image [49,50].
- The spatial neighborhood criterion uses the proximity constraint : if one pixel is close to another, so should their two corresponding pixels in the other view [51].

In the literature, most underwater dense pipelines are based on the geometric criterion [21,26,32,52,53,54,55,56]. Some studies also employed this criterion [37,39,40,41] through the use of software developed for the air environment such as the open-source software MicMac [57] or the proprietary software Photoscan (Agisoft). The spatio-temporal criterion is rarely used in this context but is nonetheless often found in structure-from-motion (SFM) strategies because of its tracking capabilities [26]. As for the spatial neighborhood criterion, this approach of quasi-dense reconstruction was mainly developed in the context of high disparity aerial imagery [58,59,60] and is practically absent from underwater work. It is used in some studies [16,37,38,61,62] through the open-source software PMVS (Patch-based Multi-View Stereo) [63] and in a recently developed pipeline for an aquatic study [64], both times in combination with the geometric criterion.

The main drawback is that water greatly amplifies the effects of rays of light passing through different diopters (water, air, housing glass, lens glass). The consequence is that it significantly weakens the *a priori* pinhole hypothesis. Apart from Servos *et al.* [56] or Drap *et al.* [65], these studies do not clearly specify whether they adapted the geometric criterion, used to densify the matching, to the underwater environment (although they adapted their three-dimensional reprojection models).

Whatever their criterion, dense matching algorithms are very sensitive to the specificities of underwater environment. Indeed, most usual pipelines have been developed to be used on static or rather rigid scenes (such as objects, or indoor or urban areas) and they encounter difficulties when faced with the dynamics and unstructured composition of natural environments [66]. Thus, moving objects (fauna or flora) are usually rejected from the reconstruction or add errors in the final cloud [67].

**Figure 2:**
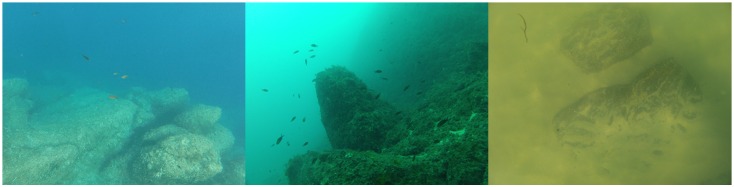
Illustration of different optical characteristics of the aquatic environment. From left to right: electromagnetic wave absorption causes elements to disappear beyond a certain distance; at 26 m deep, colors have all disappeared except in the blue-green bands; an example of turbidity in the Atlantic water.

In addition, the quality of shallow underwater images suffers from highly variable effects (such as loss of sharpness, contrast and colors, inhomogeneous illumination or changing reflectance, see Figure 2) which interfere with similarity measurements. These problems are mainly treated in the literature by preprocessing the original data [16,25,27,43,45,52,54,68]. However, this often reinforces noise and artifacts. Some studies [46,64,68] have also attempted to use multiple point detectors to increase the quantity of extracted data. Residual errors are essentially dealt with by using filters.

Moreover, the high absorption of electromagnetic waves underwater, amplified by turbidity, tends to lead to a big drop in visibility with an increase in the distance. This phenomenon could make entire parts of the images devoid of real information and which should not be reconstructed. In the literature, they are mainly removed manually from the final cloud, while some authors, like Sedlazeck *et al.* [26], have tried to reject them automatically using background segmentation.

To conclude, this survey shows that in the general context and under the constraints introduced in the preamble, the difficulties related to the dense three-dimensional reconstruction of the seabed in shallow water using a simple logistics is not fully resolved in the literature.

## 3. Sensor: Our Agile Micro-System Dedicated to Optical Acquisitions in Shallow Water

The main objective of our *in situ* optical acquisition system is to provide data enabling direct identification of individual objects of interest. To achieve this goal, acquisition must be done at the correct scale, which sets the design of the main key parameters of the system.

### 3.1. Designing the Key Parameters of the System to Map at the Scale of Individual Objects of Interest

**Figure 3:**
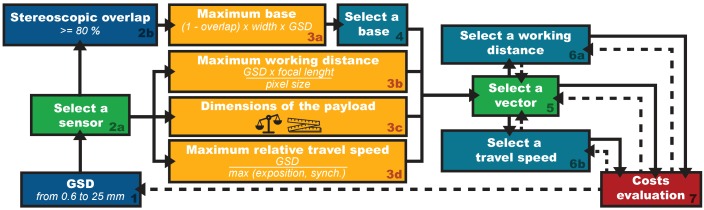
Adaptation to the underwater environment of the Extended Ground Truth (EGT) methodology, used to design the key parameters of the system to perform acquisitions at the scale of individual objects of interest. Some parameters, like the Ground Sample Distance (GSD) or the stereoscopic overlap can be pre-set by the needs of the study.

**Figure 4:**
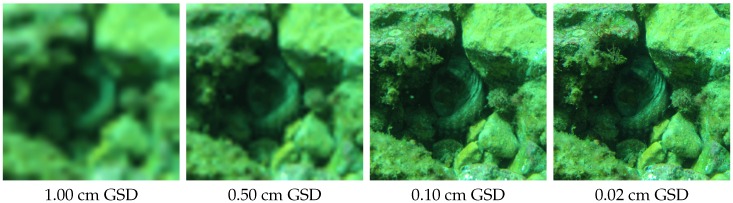
This series shows the level of detail available for the same scene according to a given GSD. To identify the octopus among the rocks, a subcentimeter GSD is needed.

The approach used in this section is a version of the Extended Ground Truth (EGT) methodology [69] adapted to an underwater environment. This methodology designs the theoretical parameters of the system from the needs of the field experts. The Figure 3 presents the flowchart we follow. Targeted objects of interest concerned by our study can be either mineral (such as rocks, pebbles or stones), vegetable (such as forests, meadows, algae or seagrasses), animal (such as reef, aggregation, corals, sea anemones, invertebrate or fish) or manufactured (such as ceramics, hulls, apparels, walls or pipelines). Their typical size ranges from centimeter to decimeter. To directly identify them, 12 to 15 pixels per object are needed [70,71,72,73]. So, the Ground Sample Distance (GSD, *i.e.*, the physical section corresponding to the side of a pixel) for our targeted objects should range from 0.6 mm for the finest analyses, to 25 mm for coarser ones (see Figure 4). To obtain the ranges for the other key parameters we use representative technical characteristics of light-weight cameras.

Ranging the GSD implies ranging the maximum distance to the target ($D_{mm}$, commonly called the height in aerial photogrammetry). Equation (1) shows their relationship, where $p_{mm}$ is the physical size of a pixel on the sensor and $f_{mm}$ its fixed focal length. Considering the magnifying effect of water [7], the maximum distance to the targeted objects is ranged from 0.5 to 4 meters for fine analyses and from 10 to 15 meters for coarse ones.

(1) $$D_{mm}\leq \frac{GSD_{mm}\times f_{mm}}{p_{mm}}$$

The distance to the target will influence the depth granularity ($Gz_{mm}$, also known as depth uncertainty) [74], that is, the ability of the system to resolve the depth of an object at a given distance. As shown in Equation (2), it is constrained by the baseline ($B_{mm}$, *i.e.*, the physical distance between the optical centers of the two stereoscopic images).

(2) $$Gz_{mm}=GSD\times \frac{D_{mm}}{B_{mm}}$$

For a given GSD, the greater the baseline, the finer the depth granularity. However, enlarging the baseline reduces the stereoscopic overlap ($O_{prct}$, *i.e.*, the common part of the scene shared by the images of a stereopair) as we can see in Equation (3) (for dual camera systems), where $W_{pix}$ is the width of the images in pixels. It is generally recommended in aerial photogrammetry to have a stereoscopic overlap of at least 60% (0.6) [75,76]. To favor dense local reconstruction conditions, a greater overlap of at least 80% (0.8) is chosen. With our previously fixed GSD, it yields a baseline from 5 to 30 cm for fine analyses, and a more relaxed one (up to 1–2 m) for coarser ones. As a result, the obtained granularity ranges from 1 to 50 mm (fine studies) and from 125 to 250 mm (coarse studies).

(3) $$B_{mm}\leq \left(1-O_{prct}\right)\times W_{pix}\times GSD_{mm}$$

The previous considerations depend only on the optical part of the sensor. However, its electronic part also constrains the system and introduces potential blurs on images. The exposure time ($Exp_{s}$) is the length of time needed by the sensor to collect enough photons and convert them to digital measurements. To prevent motion blur, the relative displacement of the observed scene to the sensor during the exposure time must be smaller than the GSD. The synchronization time ($Synch_{s}$) is the length of time separating the two acquisitions of the same stereopair. To prevent *synchronization blur*, the relative displacement of the observed scene during the synchronization time must also be smaller than the GSD. Therefore, the maximum relative travel speed of the system ($rs_{mm/s}$) has to be dynamically adapted to *in situ* lighting and dynamic conditions (Equation (4)).

(4) $$rs_{mm/s}\leq \frac{GSD_{mm}}{max(Exp_{s},Synch_{s})}$$

Average exposure time in shallow water varies around 3 ms. It can rise to 70 ms when the lighting is low or drops to 1 ms in very good conditions. As for an achievable synchronization rate, it usually stands between 500 ms and 1 ms. Therefore, in an average case, the maximum relative speed ranges from 0.1 m/s (detailed studies) to 2.5 m/s (coarse studies).

**Figure 5:**
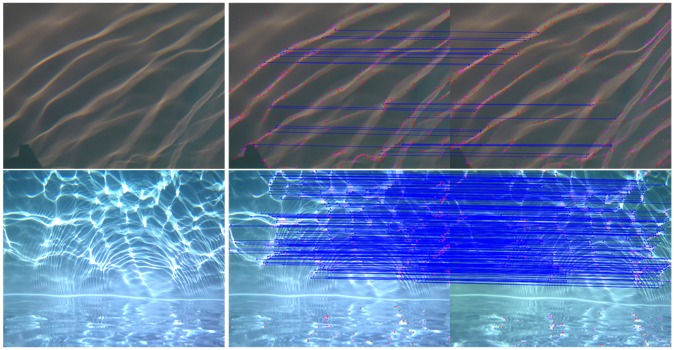
Demonstration of the impact of synchronization during the matching stage in the presence of highly dynamic movements: flickering illumination (illustrated on the left). The point detector and sparse matching algorithms used for this test are described in Section 4. **Top**, a stereoscopic pair with a synchronization time of slightly less than half of a second: 700 points detected, 375 matches, but only 26 valid; **Bottom**, a stereoscopic pair with a better synchronization time of one thousandth of a second: 700 points detected, 536 matches, 471 valid.

On our observation scale, the dynamism of the scene is no longer negligible and the use of a single sensor (temporal stereopairs) is no longer possible: two sensors are required (simultaneous stereopairs). In practice, obtaining a precise (higher than one hundredth of a second) and stable synchronization of affordable, light-weight cameras is problematic, even with an electronic simultaneous trigger. Figure 5 demonstrates how such synchronization times can be insufficient in highly dynamic situations and the impact on matching algorithms.

**Figure 6:**
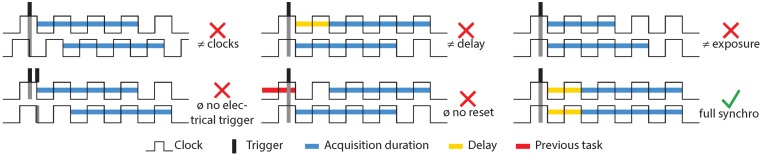
The main parameters involved in the synchronization of two cameras. Only the last case can be considered fully synchronized: a common clock for the two devices; an external electronic trigger to request acquisition; a fixed delay between the reception of the trigger and the physical action; the ability of the sensor to reset the previous action and immediately begin the new one; and finally, a fixed acquisition time common to both sensors.

**Figure 7:**
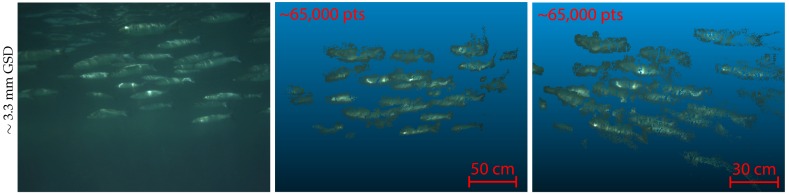
Tridimensional reconstruction of fauna becomes possible with an adequate synchronization time between cameras. Here a school of fish (mullet) taken with a synchronization time of less than one thousandth of a second (**left**) and two views of the point cloud extracted from one stereopair with the reconstruction algorithm we will present later (resolution reduced from 1280 × 960 to 700 × 525, GSD is given accordingly).

Indeed, the electronic settings of these cameras (detailed in Figure 6) that allow a truly effective synchronization are seldom accessible and need to be hacked. This situation should change with the improved availability of open-firmwares. Figure 7 shows that with an adequate synchronization time, it is possible to reconstruct in three dimensions very dynamic objects such as fauna, even with affordable, light-weight cameras. This result could be very interesting for underwater biologists who work on statistical measurements of fish [77,78,79,80,81].

Table 1 summarizes the theoretical key parameters which allow the design of the acquisition system (vehicle and payload). To be able to dynamically adapt the relative travel speed to the ever changing environmental conditions of shallow water and limit the impact of blurs, the vehicle and sensor have to work closely together. As this kind of interaction cannot be achieved with off-the-shelf micro-ROVs, we have developed our own robotic system.

### 3.2. A Micro-System to Perform on Demand Acquisition in Shallow Water Using Simple Logistics

As the acquisition system is destined to be used by the field experts, it should meet criteria such as agility, simple logistics, ease of reproducing. The operational agility of a system as well as the logistical complexity involved in its transportation and deployment are mostly affected by weight and size. As for ease of reproducing, which is important for operational flexibility and for facilitating maintenance tasks, it involves both low costs and simple manufacturing process.

**Figure 8:**
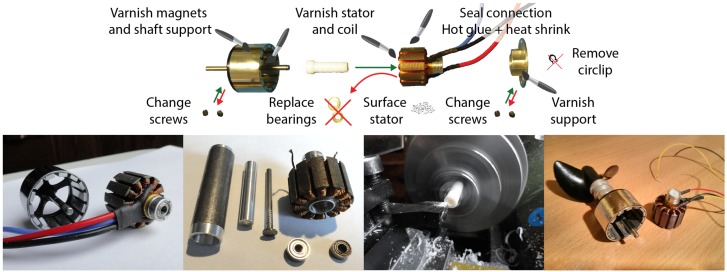
Illustration of the procedure that we conceived to marinize brushless motors so they can run in total immersion in salt water.

One of the biggest constraints on both size and weight comes from the propulsion system (propellers, motors, controllers and batteries). Indeed, most cheap sealed motors currently available (such as Seabotix) are significant in size (≳20 cm, ∼700 g) and need to be supplied by a consequent battery. To solve this problem, we adapted to underwater environment the efficient micro brushless motors we had previously used on our aerial multirotors (UAVs) [82,83]. Although the dimensions of the motors were kept (<5 cm, ∼50 g), we used a higher torque than in air (800 kV), which is more appropriate for the aquatic environment and yields a 690 g thrust. To achieve this, we created a marinization method enabling them to run in total immersion in salt water [84]. Figure 8 shows the method which includes our latest improvements following a three years of field testing. A brushless motor run barely 10 h in salt water (40 g/L) before seizing up. With our modifications, the same motor showed no sign of wear after running hundreds of hours.

Concerning sensors, among the affordable solutions that comply with the range values set in the previous section, we selected two small and complementary types of cameras: uEye and GoPro. The uEyes are low-cost professional cameras. In the literature, they have been mounted as a stereoscopic system for near real-time autonomous navigation of UAVs (Unmanned Aerial Vehicles) [85] and as a single embedded camera in some underwater projects [86] for remote control purpose. They need a computer to operate in order to trigger the shooting, retrieve the acquired images and save them. We designed all this control pipeline to optimize the use of limited computation resources of embedded units (parallel programming) and to be dynamically adaptive to the changing environmental conditions. We conducted an experimental measurement of the synchronization time: the comparison of the value of a digital counter (accurate to the thousandth of a second) on the two views shows an accuracy of three-thousandths of a second, with no drift over the time.

The second type of camera, the GoPros are popular action cameras. They are characterized by a good image quality even when compared to professional equipment [87]. They are autonomous in energy and store the images on internal memory (no need for an external computer) but they are not meant to be configured or controlled by a robotic platform. Some modification is therefore required. Indeed, some functions (such as white balance or ISO) cannot be disabled because of the actual proprietary firmware. With our modifications, we measured a synchronization time of half a second using the time-laps mode and of three-hundredths of a second when using the synchronization cable accessory (with a small drift over time).

**Figure 9:**
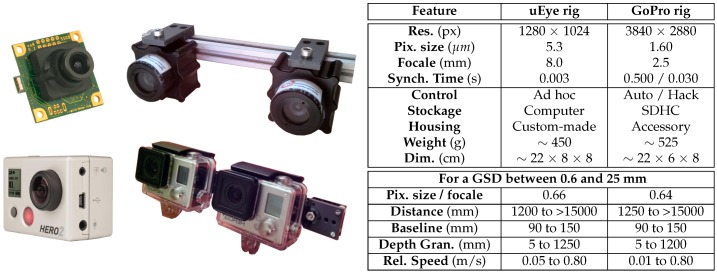
The uEye (**top left**) and GoPro (**bottom left**) cameras, the built rigs (**center**) and their main technical features (**right**).

**Figure 10:**
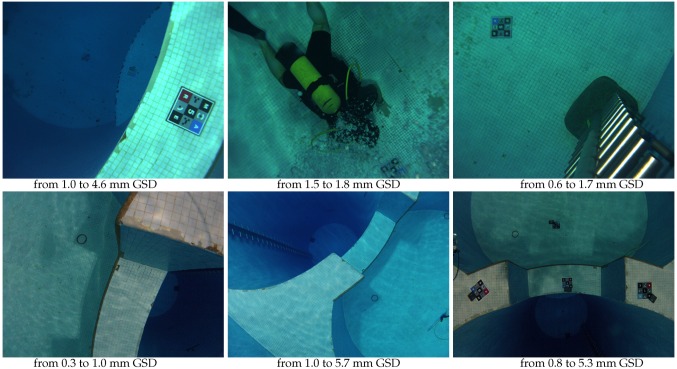
Examples of lefthand images from stereopairs acquired by the uEye rig (**top**) and the GoPro rig (**bottom**) in the diving pool.

We built custom waterproof housings for the uEyes and high-speed sealed communication cables which allow the communication with the robot. The built rigs enable the baseline to be adjusted from 9 to 15 cm with a centimetric step. An illustration of both cameras, rigs and a table summarizing the main technical features can be found in Figure 9 while Figure 10 provides sample images acquired in a diving pool. Both rigs are mounted on the vehicle. The double acquisition is very useful when access to the test areas is time limited. Indeed, their complementarity offers two different fields of views of the same scene and the setting of different baselines allows two levels of granularity to be captured in a single coverage.

**Figure 11:**
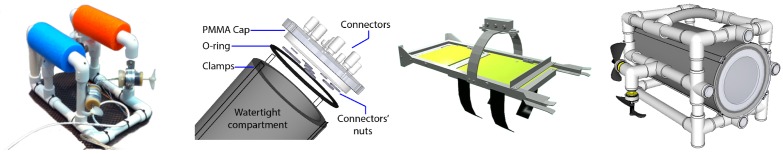
The design of our agile micro underwater robot. From left to right: our prototype of the micro pedagogical ROV SeaPerch which inspired our design; the sealing system we designed; the model of the inner frame; the three-dimensional model of our robot.

Concerning the simplicity and the ease of duplication of the system, the design of our micro-robot is inspired by MIT’s Seaperch pedagogical project [88,89] (Figure 11). Our sealing system is simple, robust and allows the housing to be quickly opened and closed anywhere without tools. It consists of a large pressure PVC (*PolyVinyl Chloride*) pipe and two machined transparent PMMA (*PolyMethyl MethAcrylate*) caps maintained together by clamps and sealed with O-rings. The inner frame is machined and locked to the cap with sealed connectors to enable communication between inside and submerged elements. A large part of our system is made up of these submerged elements to favor modularity (such as disposition, addition or removal). We designed an affordable method using hot glue and heat shrink tube to achieve interchangeable wet connections for these blocks. They are mounted on a drilled outer frame that allows air venting and water draining. It is made of PVC pipes and fittings that interlock and can be easily reconfigured. The construction of our complete structure only requires simple tools such as a cutting machine, saws or files.

For greater flexibility, we designed the system as a hybrid: it can be used in both ROV and AUV modes depending on the type of mission. To ensure the systematization of data acquisition required in scientific robotic missions, the levels of automation implemented for the AUV mode can be used in ROV mode. The experimental results of our depth control yield sub-decimetric precision. This is sufficient to obtain the adequate mean distance of observation for ensuring the targeted GSD when considering low sloping areas. The precision of our absolute travel speed control (resulting from the integrated closed-loop of brushless motors) is lower than 5%. This helps to prevent most motion and synchronization blurs.

**Figure 12:**
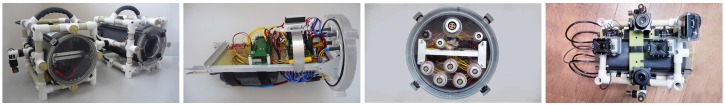
Built prototype of Ryujin. From left to right: two prototypes of Ryujin; the electronics mounted on the internal frame; the rear cap with its sealed connectors; the payloads mounted under the robot.

**Figure 13:**
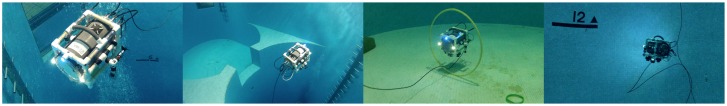
The underwater vehicle Ryujin during test sessions in the diving pool of Charenton-le-Pont.

The resulting prototype, Ryujin (*Sea Dragon*) was among the first micro hybrid robots built. Its main features are summarized in Table 2. Ryujin is presented in Figure 12 and can be seen during tests in a diving pool in Figure 13. Our prototype has currently been tested for about fifty hours in a diving pool over a period of three years. Ryujin also successfully performed autonomously in a port during two consecutive editions of the SAUC-E competition, a European student challenge for autonomous underwater vehicles.

This section concludes how stereoscopic pairs at the scale of individual objects of interest are acquired by our system using simple logistics. The next section is dedicated to how our system robustly processes these underwater data to extract their three-dimensional information.

## 4. Post-Processing Pipeline: Retrieving the Three-Dimensional Information from Underwater Stereopairs

As seen in Section 2, three strategies can be used to create dense point clouds. Those using geometric or spatio-temporal criterion inherently assume that corresponding pixels have a high similarity score. Underwater, this systematic approach often fails in non textured areas resulting from high absorption. Indeed, the pixels of such areas yield high similarity scores, but matching them makes no sense. Because of its local adaptation abilities to detect these cases, we have chosen to rely solely on the spatial neighborhood criterion and designed an algorithm that propagates the matching from the most reliable areas (the ones that are the most textured) to the areas that are less so.

**Figure 14:**
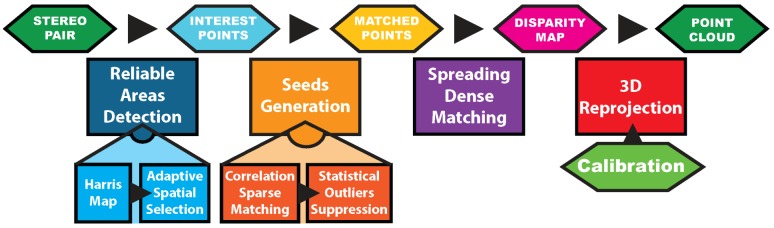
Overview of our underwater three-dimensional reconstruction algorithm.

An overview of our algorithm is shown in Figure 14. It starts by identifying the most textured areas on the images. Pixels representative of these areas are then matched inside each stereopair to form a robust subset called seeds. Afterwards, the matching is expanded in the neighborhood of the seeds to enable a reliable densification in the underwater context. Finally, all matched pixels are reprojected using the camera model to form the three-dimensional point cloud. In this section, we describe all these steps in details.

### 4.1. Robust Identification of the Most Textured Areas in Underwater Images

The first step of the three-dimensional reconstruction algorithm is to extract a subset of pixels representative of the textured areas to form the seeds. Points of interest are naturally good seed candidates as they present distinctive features and therefore allow a reliable match. Many aerial interest point detectors have been used underwater. These include SIFT [90], SURF [91] and Harris [92]. Méline *et al.* [46] and Skarlatos *et al.* [38] showed that SIFT and SURF detectors-descriptors are particularly sensitive to speckle noise and vignetting effect which greatly weaken their performance on underwater images. Conversely, the Harris detector focuses on textured areas and marks their breaks. For these reasons, we decided to use the Harris detector to identify the seed candidates.

**Figure 15:**
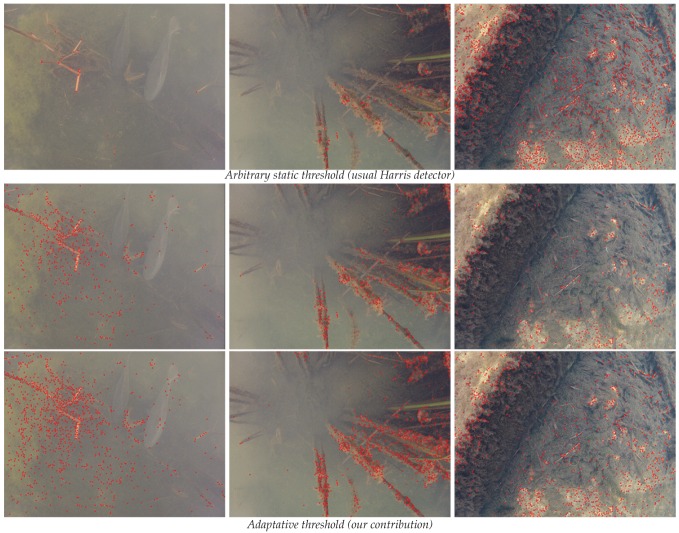
Adaptation of the Harris detector to the variable quality of underwater images.These images were all taken in a lake by a single camera in close proximity to each other (<5 m) and within a few minutes. The detected Harris points are represented by red circles. The static threshold of the usual algorithm is arbitrarily set at 189: we obtained only 20 points on the first image, 200 on the second and 2078 on the third. For the first series with our adaptive thresholding, the target interval is set at [500–600] and we obtained 500 points for the three images with thresholds that converged at 108, 167 and 231. For the second series, the interval is set at [1000–1100] and we respectively obtained 1023, 1000 and 1004 points for final thresholds at 95, 144 and 213.

However, the number of points of interest found by the Harris detector is highly sensitive to the quality of the input image, as we can see on the images at the top of Figure 15. As the number of seeds used to start the spreading strongly influences the quality of the final reconstruction, we suggest changing the behavior of the algorithm. Our modification allows the number of detected points to be monitored more independently of the quality of original images by using a closed-loop approach.

As a reminder, the first step of the Harris detector in its usual version gives a measurement of interest to each pixel, called the Harris map. The second step deals with the selection of the feature points. An arbitrary, universal and static threshold is used to separate the pixels of interest, (*i.e.*, with a high variability of texture, that is, a high value in the Harris map) from the other pixels. Finally, of these, only local maxima are selected over small areas to form the final set of Harris points.

This second step holds the key to controlling the number of extracted points. Simply thresholding the histogram of the Harris map at the required number would not supply an adequate solution as it does not take into account the spatial distribution of the selected points. Therefore, we propose another kind of modification to reach our goal. A first initial threshold is set as a percentage of the maximum value of the Harris map to fit to the real measurements. Then, a first set of spatially selected interest points is computed. If the number of points in this set is not included in the desired range of the number of interest points, then a new threshold is calculated and the process loops. The decimation part that extracts feature points from the Harris map (thresholding and selection of local maxima) is repeated until the obtained number of interest points complies with the request.

**Figure 16:**
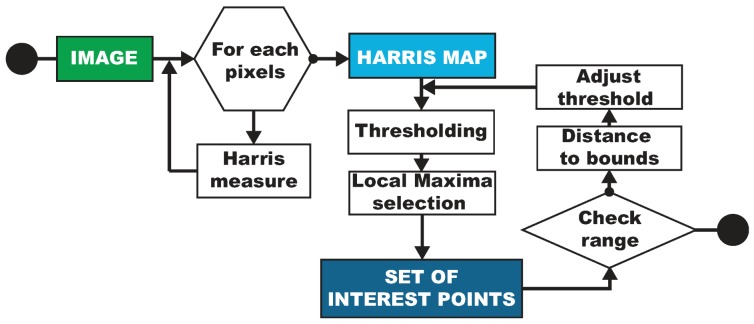
Flowchart of our modified Harris detector algorithm dedicated to the underwater environment.

To converge as quickly as possible towards the objective, the distance between the obtained number of points and the boundaries of the target range is calculated. The value of the new threshold is adjusted in proportion: if we are far from the goal, a huge modification will help to get closer effectively. Conversely, a small one will prevent oscillations around the target range. This closed-loop approach is inspired by proportional correctors used in the field of control and automation [93]. Two examples of our adaptive thresholding results are shown in Figure 15. The flowchart of our modified Harris algorithm is detailed in Figure 16. At the end of this step, we have acquired a subset of pixels representative of the most textured areas of each image of a stereopair.

Our adaptive version of the Harris detector belongs to the same algorithmic class as the usual one. The selection step is of linear complexity. To keep the number of iterations of this step as low as possible, we suppose that temporarily close images have a high probability of having a similar quality. So, we use the previously calculated threshold as the initial value to process the following image. On a representative set of images, we observe that, in practice, the convergence is obtained in less than five iterations. The computational time difference (*i.e*., number of processor cycles) between the usual Harris detector and our adaptative version is less than 5%. A limit to the adaptive ability of the algorithm must be imposed: the image is rejected if too many image pixels are selected as points of interest.

### 4.2. Creation of a Reliable Set of Seeds

To fulfill the set of seeds, the Harris points detected in the two images of the stereoscopic pair must be matched. A correlation measure is used to evaluate the similarity. Potential matches whose value is too low are rejected. To be efficient, this operator supposes that there is little to no rotation and only small translations between the two views. This is our case, because our stereoscopic rig is rigid, synchronized and has a small baseline. By default, the fast SSD (Sum of Squared Differences) criterion is used. It gives good results on submarine views with no strong color differences. When the matching percentage is too low, the algorithm switches to the more robust but more complex ZNCC (Zero-mean Normalized Cross-Correlation) criterion. The size of the correlation window is important: small ones do not have enough information to obtain a reliable discrimination whereas big ones are time consuming. In our case a 13 × 13 window offers the best compromise on average.

**Figure 17:**
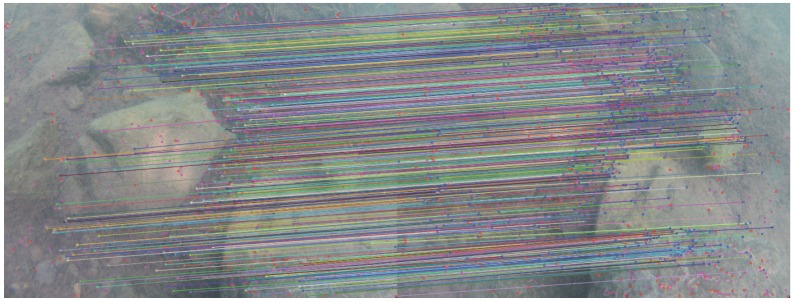
Result of the seed generation step of our algorithm on an underwater Stereopair (Lac Pavin, Auvergne): out of 969 initial interest points, 517 are successfully matched (53%).

**Figure 18:**
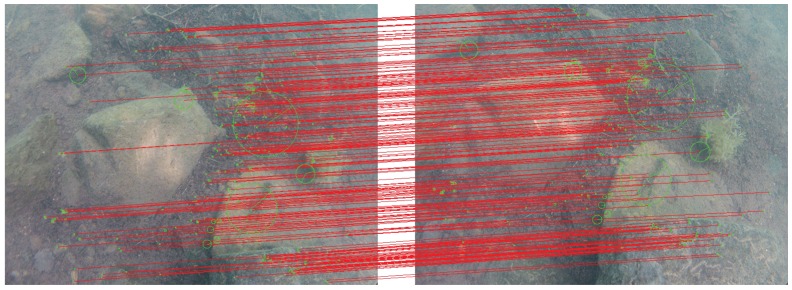
Result of seed generation using an implementation of the SIFT algorithm [94] (same stereopair as Figure 17): out of 954 initial interest points, 263 are successfully matched, (*i.e.*, 27% points).

Figure 17 shows the results of the seed generation step of our algorithm on an underwater stereopair: 53% of the detected interest points are matched. In comparison, Figure 18 presents the results of an implementation of a sparse matching algorithm based on the SIFT detector-descriptor on the same stereopair. To facilitate the comparison, the target range of our algorithm was fitted to the number of interest points found by the SIFT algorithm. As a previous result, our algorithm matches almost 50% extra points than SIFT for this stereopair.

Quantity alone is not sufficient to ensure the quality of dense reconstruction. It is also necessary to ascertain the reliability of the seeds, that is, of the sparse matching. Filtering algorithms classify each matched pair as inlier (reliable information) or outlier (error). To achieve this without relying on the geometry, we designed a filtering algorithm based on a statistical criterion applied over the local vector flow.

A matched vector is a useful way to spatially represent the relationship between two matched points and is defined by their coordinates placed in the same image plane. For each matched vector ${\vec{v}}_{i}$, our filtering algorithm starts to extract the local vector flow, which is the subset of matched vectors in the neighborhood of ${\vec{v}}_{i}$. Then it computes the number of matched vectors of the local flow that have similar norm and direction. If this number is too low, then ${\vec{v}}_{i}$ is considered an outlier and is removed from the list of candidate vectors. Otherwise, as an inlier, it is added to the list of seeds.

**Figure 19:**
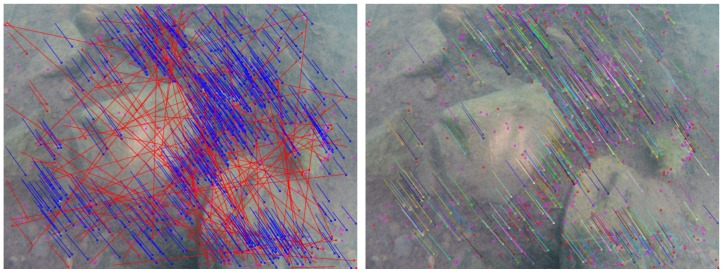
Illustration of our filtering step based on a statistical criterion applied over the local vector flow. Out of the 969 detected interest points, 660 initial matched vectors have been formed. Then, all vectors that are not consistent with the local flow are rejected (143, in red) and the others (517, in blue) form the list of reliable seeds (78% of the matched points are inliers).

Isolated pairs are systematically eliminated, which is preferable as the areas they represent would not provide a reliable spread. Figure 19 shows a result of our filtering algorithm: more than 75% of the initial matched vectors are valid. All outliers are efficiently filtered, and, as expected, some isolated inliers are excluded by the statistical criterion. For this test, they represent less than 2% of the outliers. If necessary, one way to reduce these false outliers is to increase the desired number of initial interest points.

To assess our seed generation algorithm and produce some general statistics, we created an underwater image database. This database is composed of about 100 underwater stereoscopic pairs. The images were taken in various lighting conditions and in various environments (such as lakes, rivers or seas). The images are subsampled from 3840 × 2880 to 700 × 525 pixels.

**Figure 20:**
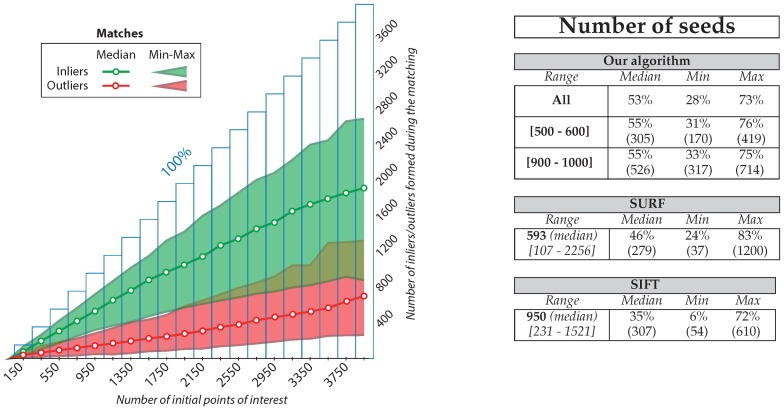
General statistics obtained from a sample of 200 images taken in six different stretches of water. Left, the chart provides the statistical results of our algorithm and right, the statistical results are compared with those obtained by SURF and SIFT on the same sample images.

As seen before, a key point of our algorithm is that it allows a target number of interest points to be selected. This ability really distinguishes it from more usual algorithms dedicated to sparse matching. Therefore, we compute the number of inliers (seeds) and outliers obtained according to the number of interest points requested (target ranges). For each range from [100–200] to [3900–4000], computations are done on the whole database. The results are shown as a graph in Figure 20.

The ratio of inliers *versus* outliers is stable (±4% only over all the ranges) even when the number of interest points is very low or very high. In general (median), our algorithm can extract 53% of seeds from the initial interest points (73% in the best cases and 28% in the worst cases). Furthermore, only 23% of the initial matches become outliers (7% in the best cases and 46% in the worst cases) and no false inliers have been found. Given these results, we can consider that our algorithm is robust to the number of interest point requested.

Implementations of SIFT and SURF detectors-descriptors available in the OpenCV library have been used to process the image database. The results obtained are presented in the tables of Figure 20. Overall, SURF performs a little bit better than SIFT (46% *versus* 35% median value), but we must note that SURF is very irregular in its results and beyond 1000 points of interest the median rate falls below 40%. The filter also often lets through some outliers (3–5). Conversely, SIFT is more stable and unfiltered outliers are less common (one or two occasionally). However, it presents a low ratio of inliers compared to the number of interest points and this ratio further decreases with the increase in the number of points.

To allow the comparison of the results, the median number of interest points detected by SURF or SIFT is used to set the target range of our algorithm. Our algorithm obtains a higher median percentage of seed generation: 55% *versus* 46% (SURF) and 55% *versus* 35% (SIFT). To conclude, our algorithm generally yields better results and is much more consistent in its behavior.

### 4.3. Spreading of the Matching and Automatic Exclusion of Areas without Information

Once the seed generation is done, the next step of our reconstruction algorithm is to recursively spread the matching in the neighborhood of these seeds in order to produce a dense disparity map. As seen before, a seed is a couple of matched pixels: $S_{L}$ in the left view and $S_{R}$ in the right one. The neighborhood of each seed is independently studied and is used as a local starting point for the growth of its own region. Let us call $W_{L}$ a square 5 × 5 window centered on $S_{L}$ and $W_{R}$ the corresponding one on $S_{R}$. These two windows describe the neighborhood of a seed. Each pixel $P_{WL}$ of $W_{L}$ is individually evaluated to search for a corresponding pixel. For a given pixel $P_{WL}$, which has not yet been matched, a matching candidate is sought in a 3 × 3 window, centered on the equivalent pixel $P_{WR}$ of $W_{R}$. This yields nine candidate pixels, whose similarity to $P_{WL}$ is assessed by a correlation measurement. If the best score is not too low, the corresponding candidate is matched with $P_{WL}$ and the color information is saved. Their disparity is calculated and then refined at a subpixel level to enhance the depth granularity of the system. This interpolation is performed by seeking the exact position of the correlation paraboloid peak. The subpixel disparity is saved to complete the disparity map. A new match can be better than one already saved in the disparity map because search windows overlap (candidate pixels are side by side). In this case, the new $P_{WL}$ corresponding pixel is saved and the former is released and may be evaluated again if it falls in a new $W_{L}$.

Each point matched in the neighborhood of a seed becomes a new seed to spread the matching in its own neighborhood. As long as there are seeds to study, the regions will continue to grow in this way. We then distinguish two possible configurations: collision or expansion. Indeed, regions near each other will meet soon and stabilize around a border. Still unmatched pixels $P_{WL}$ of current studied windows $W_{L}$ can replace former matches if they give a better result. This will refine the border, but the situation will stabilize because $P_{WL}$ pixels that are already matched are not reconsidered. The respective spreading of these regions in this direction will then be blocked. Conversely, if there is no collision, the regions may extend as long as matches are found. The more we move away from the original seeds, the more we depart from the reliable textured areas. Indeed, nothing matches more easily than two points in a somewhat "uniform" area. We therefore limit the spreading within a radius around the original seeds, and areas without real information are thereby automatically excluded from the final reconstruction. An optimum situation is obtained with a sufficient number of seeds and a small radius of expansion.

**Figure 21:**
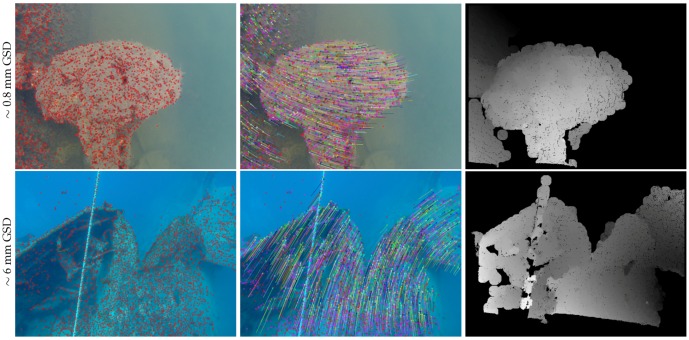
Matching densification by propagation and automatic exclusion of areas without real information. Two stereopairs taken in the lake of Plage Bleue, France (**top**) and over the Thistlegorm shipwreck, Egypt (**bottom**). **Left**: identification of the most textured areas (modified Harris points); **Center**: seed generation (vector flows); **Right**: dense matching by spreading (disparity maps). On the disparity maps, the excluded areas are in black. The disparity is represented in grayscale: the higher the value (lighter gray), the greater the disparity, the closer to the camera.

**Figure 22:**
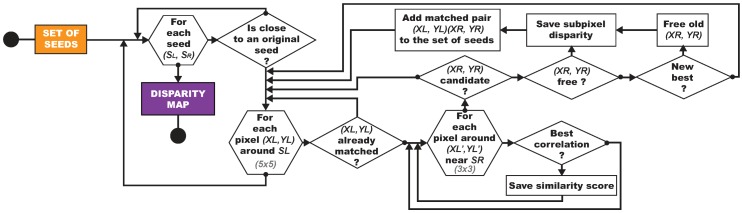
Flowcharts of our dense matching by propagation.

Figure 21 presents two examples of the densification step demonstrating the automatic exclusion of unreliable areas and shows the obtained disparity maps. The flowchart of our matching densification algorithm is provided in Figure 22.

### 4.4. Reprojection and Three-Dimensional Point Cloud

To finalize the process and obtain a dense and colored point cloud, we have to reproject each pixel of the disparity map in the camera frame. A pinhole model is used to simplify the perspective transformation performed by the sensor when recording the scene information as an image. This model uses several parameters of the sensor (such as focal length, optical center positions, radial and tangential distortion parameters, pixel ratio, *etc.*) which are usually estimated by calibration. However, in our context, the huge differences in physical propagation of light in water, glass and air create significant effects that invalidate the pinhole model. This model can nevertheless yield a satisfactory approximate reprojection, provided that all these new diopters are considered as additional sensor lenses [21,37,38,45,95,96].

Indeed, by artificially increasing the focal length of the sensor, the image plane is virtually moved backwards, thereby redressing all re-projected rays to compensate the bending effect of the diopters. Furthermore, by altering the radial distortion coefficients, the focal length can be locally adjusted to handle the differences in the refractive index regarding the angle of incidence: this creates an artificial curvature of the image plane. The drawback is that this approximation is accurate only for a given distance of observation.

From an operational point of view, these artificially altered parameters can also be estimated by calibration, but this has to be done in the presence of the diopters. That is why the calibration must be performed *in situ*, directly in the waters of the mission. For our *in situ* calibration, we used a flat asymmetric chessboard target (45 × 45 cm, 9 × 10 squares). The procedure of acquiring calibration data is the same as in air, except that the distance to the target must be constant and equal to the distance of observation required by the study.

**Figure 23:**
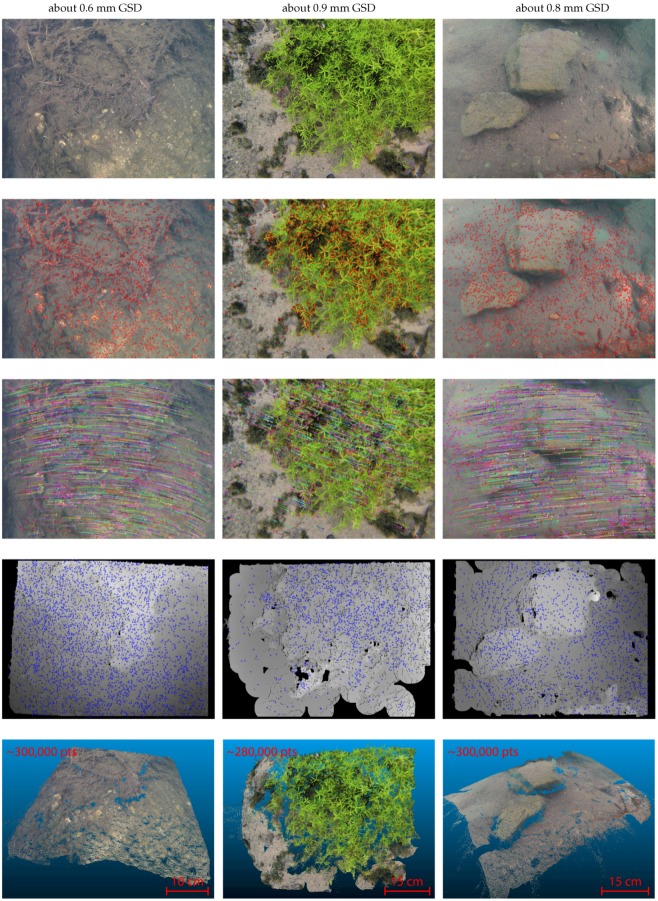
Illustration of the processing pipeline on sample images acquired in lakes with a single GoPro (no rig, small displacements, resolution reduced from 3840 × 2880 px to 700×525 px and GSD given accordingly). From top to bottom: original lefthand image of the stereopair, identification of the most textured areas (modified Harris points), seed generation (vector flows), dense matching by spreading (disparity maps), and finally three-dimensional reprojection (colored point clouds).

Figure 23 illustrates all the steps of our three-dimensional reconstruction algorithm on three examples. As we used a fixed baseline (rigid rig), the *in situ* calibration needs only to be done once per mission. Furthermore, as the cameras are in the same plane with parallel optical axes, this simplifies the geometry of the system and therefore simplifies the calculations used for the reprojection [97]. In addition, the knowledge of the baseline (distance between the two cameras) allows the reconstruction of the scene at the right scale factor.

## 5. Results of Field Missions in the Mediterranean Sea and Analysis

**Figure 24:**
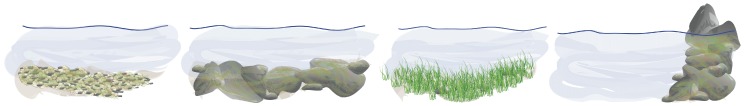
Illustrations of the diversity of the seabed in the study area at Cap de Nice.From left to right: screes, rocks, seagrasses and cliffs.

**Figure 25:**
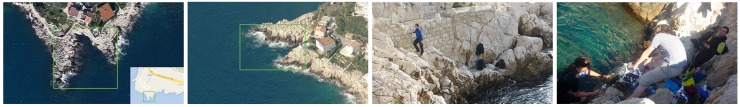
The study area at Cap de Nice is marked by the green square on both satellite image (Google Maps**©**) and airborne view (Bing Maps**©**) and two photographs on the right show the launching platform below the Sea Trail.

In this section, we report on how our work stood up to experimental field conditions. This analysis completes the results presented in the previous sections. This location presents many interesting features like clear water or varied seabed which offers both texture and relief (such as screes, rocks, seagrasses or cliffs, as seen in Figure 24). In addition, sufficient depths (4–15 m deep) are accessible near the coast without the use of a boat. All these conditions are representative of the experimental setting exposed in the preamble. The mission took place over three days in August 2013 and two days in late October 2013. The size of the study area was approximately 1000 m${}^{2}$, and is shown in Figure 25.

### 5.1. Equipment of the Field with Landmark Patterns

Using markers on the field as control points is a common practice in photogrammetry as well as in computer vision. By conducting real measurements on these markers, such as depth or position, they enable the establishment of a scaled reference frame. The latter is used to control the accuracy of the three-dimensional reconstruction. Concerning these markers, underwater literature shows two main possibilities: rules (alternating bands of color representing known distances) and archaeological markers (labels with numbers and colors) [98].

These solutions are not optimal for computer vision algorithms. Indeed, as rules are a periodic repetition of the same pattern, it creates indecision in an automatic process. This problem does not occur with archeological markers as they are all different, but their spatial extent is lacking, which do not ensure their detection on images.

**Figure 26:**
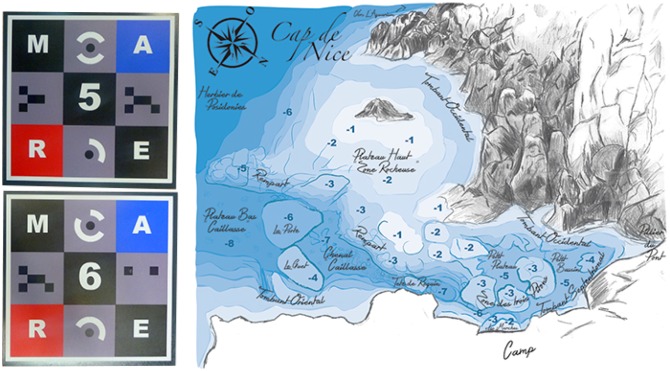
Left, the underwater landmark patterns we designed and used to equip the study area; and right, the sketch of the study area realized from *in situ* notes.

The landmark patterns we designed (Figure 26, left) are a compromise of what is done in aerial and computer vision work [99] and were inspired by the feedback given in Skarlatos and Rova [100]. Our landmarks are large (20×20 cm) to be easily detected on images and composed of colored tiles acting as scale factors (equivalent to rules). Each landmark has a unique pattern of numbers, targets and colors which facilitate its identification together with its spatial orientation. After feedback from the first mission, the original white tiles were replaced by gray ones to avoid overexposure problems with the cameras.

We observed from field experience that a compromise between spatial and vertical equirepartion of the landmarks gives a wealth of information on the three-dimensional morphology of the scene. In our case that means around thirty landmark patterns.

The ground truth measurements from the landmark patterns supplemented by *in situ* notes made it possible to draw rough maps of study areas. This summary spatial information is very useful for operational decision making (see Figure 26, right).

### 5.2. Analysis of the Obtained Results

**Figure 27:**
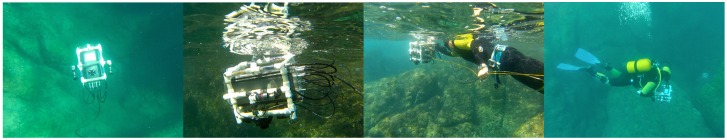
For the *in situ* acquisition, the robot is equipped with both rigs (uEye and GoPro). It is used in ROV mode with an automatic depth control and is accompanied by a diver who oversees the operation.

**Figure 28:**
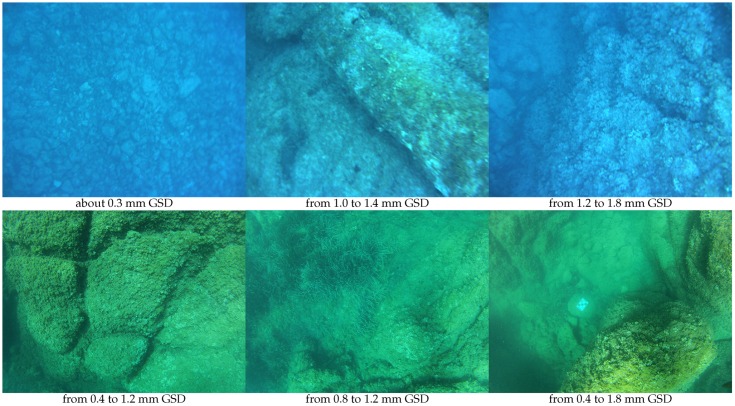
Examples of lefthand images of stereopairs acquired by the uEye rig (**top**) and GoPro rig (**bottom**).

**Concerning the acquired data,** the system was used in ROV mode with an automatic depth control and was systematically accompanied by a diver (Figure 27). The uEye and GoPro rigs were used for the acquisition. Sample images acquired by both sensors are shown in Figure 28. The primary objective to visually identify individual objects of interest is successful. The experimental GSD can be measured on images containing a landmark. The knowledge of its depth combined with the depth measurement of the robots allows the distance between the sensor and the landmark to be estimated, and so the theoretical GSD. As expected, the experimental and theoretical GSD are close: the residual difference can be explained by our error in depth measurements (±5 cm).

**Figure 29:**
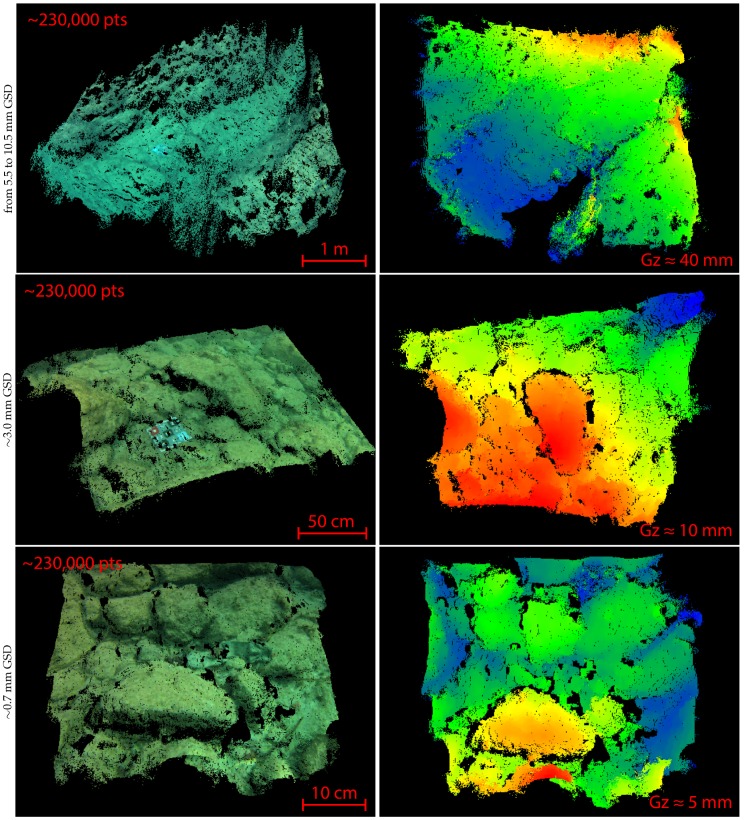
Examples of three local point clouds of the seabed, each extracted from one stereopair (two views). Left, the clouds in real color and right, the same clouds in scale color (the reprojection is relative to the cameras, the closer the point to the camera, the warmer the color). The image resolution has been reduced from 3840 × 2880 px to 700 × 525 px (the GSD is given with respect to the sub-sampled images).

Approximately 20,000 stereopairs were acquired over the whole area. However, numerous acquisitions in low light conditions are blurred. So, the closed-loop allowing to adapt the vehicle speed to the sensor needs has to be improved. We extracted several dense point clouds from this dataset, some of which are illustrated in Figure 29. These results were used to analyze the performance of our post-processing pipeline.

**Concerning the robustness to the quality of the input images,** the various point clouds we produced show that the adaptive threshold step we added in the Harris detector is crucial. Indeed, on the images we acquired under quite similar conditions, the final thresholds found by our algorithm for a given target range of interest points vary in an interval of 20%.

**Figure 30:**
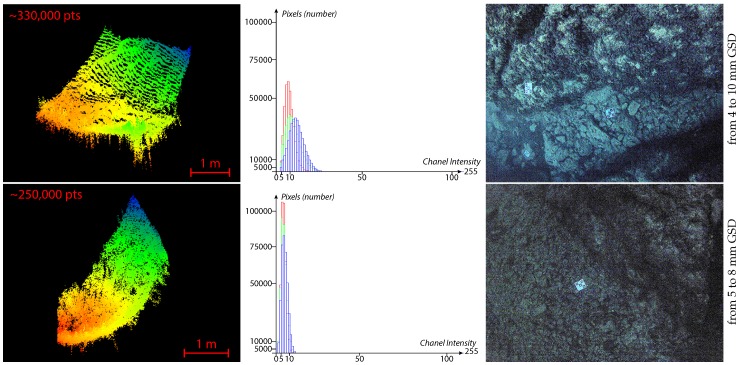
Test of the limits of our adaptive thresholding with two stereopairs acquired in very low light conditions. Left: the two point clouds obtained from the original stereopairs (no preprocessing). Center: the histograms of the input images show that all their dynamics are concentrated on less than 12% of the full range (0–255) for the first, and less than 6% for the second. Right: the lefthand images of the stereopairs with a manually greatly enhanced contrast for the purpose of illustration.

Furthermore, we acquired images with very low light conditions (close to sunset time) to test the limits of our adaptive thresholding. The input images have a narrow spectrum as all their dynamics are contained within 15% of their full range [0–255]. The adaptive thresholds converge to a value about 80% lower than that of the general case. The extracted interest points are still reliable as we can see on the obtained point clouds, shown in Figure 30.

**Concerning the density of the created point clouds,** our results show that our algorithm allows the dense matching of 60% to 85% of the pixels of the images considering stereoscopic pairs with an overlap rate between 70% to 90%. The measured density of the point clouds is consistent with the GSD of the acquisitions. As an example, measurements on the first two point clouds presented in Figure 29 show that we obtain about 1–2 points per square centimeter for a corresponding GSD between 7 mm and 5.5 mm for the first cloud, and about 6–7 points per square centimeters for a corresponding GSD of about 3 mm for the second cloud.

**Concerning the accuracy of the created point clouds,** as it is related to the scale of the acquisition, we will evaluate it with respect to the GSD. Reconstruction errors come from multiple sources such as matching (accuracy of the interpolation), calibration (estimation of the sensor parameters) or reprojection (approximation of the model or significant difference with the calibration distance). Quantifying these errors requires comparing the numerical metrics of the clouds to corresponding ground truth metrics. We have performed these *in situ* measurements on characteristic objects such as rocks using a measuring tape. The results obtained, some of which are shown in Table 3, lead us to estimate an accuracy less than two times the GSD (a<±2×GSD).

We should discuss some points about the relevance of such assessment. *In situ* measurements are difficult to carry out with a great precision [98,101]. Indeed, the accuracy of the measuring tool in itself is only ±1 mm. Moreover, the greater the measured distance, the more inaccurate it is, because of the issue to maintain the tape tension or to stay in the horizontal plane. We estimate an *in situ* measurement error of about 2% of the measured distance. When ground truth inaccuracy becomes much larger than the average inaccuracy of the reconstruction, the measurements cannot be considered significant for assessing the precision of the reconstruction. In addition, another difficulty is that it is difficult to rely exactly on the same anchor points between the actual and numerical measurements.

**Figure 31:**
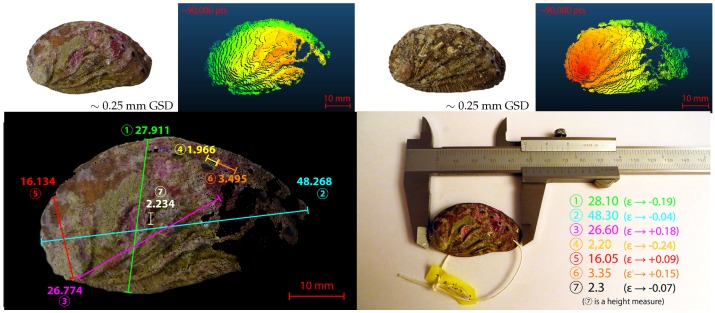
Assessment of the accuracy of our reconstructions from ground truth measurements. (**Top**), two abalone shells (*Haliotidae*) found in the study area, of about 5 × 3 centimeters digital measurements, and their respective point clouds (scale colors). (**Bottom**), digital measures on the point cloud (color mode) of the first abalone shell (**Left**) and actual measures on the object (**Right**). The difference is about ±0.2 mm, *i.e.*, about one time the GSD.

Therefore, we performed another set of measurements on small objects. Indeed, this reduces the ground truth error because we can use a more accurate measurement tool (±0.05 mm). On the other hand, it allows better control of the anchor points as we can extract the studied object from the field. To be relevant, these objects should have structural and physical characteristics (such as shape, texture or color) comparable to that of the objects composing our study areas. The chosen objects are abalone shells and Figure 31 shows the three-dimensional reconstruction obtained and the small set of actual measurements we have conducted. The difference between the numerical estimated distances and the actual ones are about once the GSD (a<±1×GSD), which is coherent with the *in situ* results.

**Figure 32:**
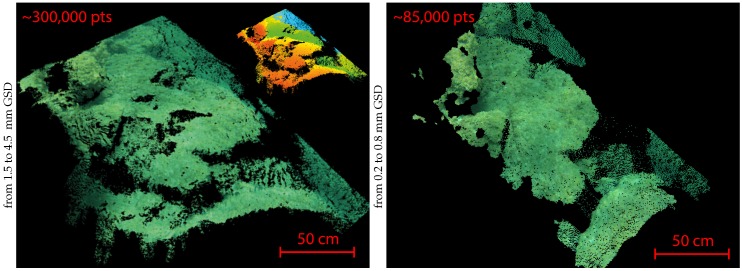
Two-view and multi-view reconstruction of a same area. Left, a local point cloud extracted from two subsampled views (700 × 525 px) with our algorithm (the scale-colored cloud is shown in the inset to give a better idea of the relief). Right, a point cloud extracted from 18 views in full resolution (3840 × 2880 px) with the multi-view reconstruction software MicMac [57].

Figure 32 shows that the point cloud obtained by our pipeline from 2 subsampled views is consistent with that obtained by the MicMac multiview software from 18 views in full resolution. The number of points obtained by our algorithm is three times higher than that of the MicMac (even if we consider the suppression of the unreliable points on the border of the point cloud) despite using a lesser amount of information. However, as expected, given the higher number of points of view used, the final MicMac result shows a more regular cloud with lower occlusion areas.

## 6. Conclusions and Future Work

In this article, we have discussed the problem of obtaining local dense three-dimensional reconstruction at the scale of individual objects of interest in shallow water using light-weight tools. We transversely address the problem on both acquisition and image processing. To efficiently acquire stereoscopic pairs on demand on small shallow water areas using simple logistics, we have designed one of the smallest agile and replicable acquisition systems: a hybrid underwater micro-robot equipped with modular optical stereo-rigs. To achieve a dense matching in the underwater environment, we proposed robust solutions to the relative movements of the scene (synchronization and travel speed control), to the versatile quality of the acquired images (adaptive thresholding) and to the strong light absorption that makes entire portions of an image completely devoid of information (robust detection of textured area and propagation strategy). Finally, we presented an analysis of the results obtained during two short acquisition campaigns in the Mediterranean Sea.

Future work will involve the registration and merging of our dense local clouds to obtain a coherent representation of a whole underwater area. We are also currently working on a method to facilitate the underwater calibration procedure and improve the estimation of our intrinsic parameters.

## Figures and Tables

**Table 1:** Summary of the key parameters needed to design the system given the GSD required by our study and the common parameters of affordable, light-weight cameras. Picking a sensor will set the parameter values within these ranges.

Ground Sample Distance	${\mathrm{GSD}}_{\mathrm{mm}}$	0.6 mm	25 mm
		*(Fine Analyses)*	*(Coarse Analyses)*
Maximum distance of observation	$D_{\mathrm{mm}}$	500 to 4000 mm	10,000 to >15,000 mm
Baseline (for a 80% overlap)	$B_{mm}$	50 to 300 mm	1000 to >2000 mm
Depth granularity	$Gz_{mm}$	1 to 50 mm	125 to 250 mm
Maximum Relative Speed	$Rs_{mm/s}$	100 mm/s	2500 mm/s

**Table 2:** Main features of our hybrid micro-robot.

*Ryujin* Underwater Vehicle				
**Size**	20 × 20 × 30 cm	**Depth rating**	100 m
**Weight**	9 Kg		(tested up to 30 m)
**Autonomy**	∼2 h	**Depth control accuracy**	∼5 cm
**Cost**	∼2000 €	**Absolute speed control accuracy**	∼5 %

**Table 3:** A sample of measurements performed *in situ* on rocks (ground truth) with a measuring tape and their corresponding measurements on the point clouds extracted at a given GSD. The accuracy is less than twice the GSD. Considering our very close observation scales, the imprecision on the actual measurements of great distances becomes much larger than the average inaccuracy of the model, thus these ground truth measurements cannot be considered significant for assessing the precision of the reconstruction (see the fourth line of the left table).

GSD	Ground T.	Cloud Meas.	Error	GSD	Ground T.	Cloud Meas.	Error
*(mm)*	*(mm)*	*(mm)*	*(mm)*	*(mm)*	*(mm)*	*(mm)*	*(mm)*
10	875	862.7	−12.3	0.8	433	434.1	1.1
10	298	307.2	9.2	0.8	427	427.8	0.8
10	49	42.3	−6.7	0.8	350	349.1	−0.9
8	1273	1248.6	−24.4	0.6	220	219.5	−0.5
3	732	734.2	2.2	0.6	170	170.8	0.8
3	218	216.7	−1.3	0.6	113	112.9	−0.1
